# Hydraulic trade-off and coordination strategies mediated by leaf functional traits of desert shrubs

**DOI:** 10.3389/fpls.2022.938758

**Published:** 2022-10-31

**Authors:** Jianqiang Huo, Yafei Shi, Jiajia Chen, Hongxia Zhang, Li Feng, Yang Zhao, Zhishan Zhang

**Affiliations:** ^1^Shapotou Desert Research and Experiment Station, Northwest Institute of Eco-Environment and Resources, Chinese Academy of Sciences, Lanzhou, China; ^2^University of Chinese Academy of Sciences, Beijing, China

**Keywords:** leaf hydraulic traits, trade-off, coordination, desert shrubs, leaf minimal water potential

## Abstract

Desert shrubs play important roles in desertification control and vegetation restoration, which are particularly affected by droughts caused by climate change. However, the hydraulic strategies associated with hydraulic functional traits of desert shrubs remain unclear. Here, eight desert shrub species with different life forms and morphologies were selected for a common garden experiment at the southeast edge of the Tengger Desert in northern China to study the hydraulic strategies mediated by leaf hydraulic functional traits. Diurnal leaf water potential change, leaf hydraulic efficiency and safety, hydraulic safety margin, hydraulic capacitance, and water potential and relative water content at the turgor loss point were observed to significantly differ among species, suggesting that leaf hydraulic functional traits were strongly associated with species even when living in the same environment. Additionally, shrubs with greater leaf hydraulic efficiency had lower midday leaf water potential and leaf hydraulic safety, suggesting that leaf hydraulic efficiency had a strong trade-off with hydraulic safety and minimum leaf water potential, whereas there was also a coordination between leaf hydraulic safety and the leaf minimal water potential. Moreover, shrubs with higher leaf hydraulic capacitance had greater hydraulic safety margins, indicating coordination between leaf hydraulic capacitance and hydraulic safety margin. Overall, this study indicated that minimal daily leaf water potential, as an easily measured parameter, may be used preliminarily to predict leaf hydraulic conductivity and the resistance to embolism of desert shrubs, providing critical insights into hydraulic trade-off and coordination strategies for native shrubs as priority species in desert vegetation restoration and reconstruction.

## Introduction

Revegetation is one of the most effective ways to control desertification and to promote ecological restoration in arid and semiarid regions (Li et al., 2004). To ameliorate desertification, the Chinese government started a series of ecological construction programs in the 1950s (Chu et al., 2019). Among them, the Three-North Shelterbelt Program (TNSP) spanning about 4.07 × 10^6^ km^2^ of Northern China plays an important role in restoring the environment, e.g., water and soil conservation, serving as a windbreak, and promoting sand fixation (Zhang et al., 2021). Generally, desert shrubs are often used as pioneer species in ecological restoration owing to their high resistance to extreme environments and their positive role in altering surface wind and improving soil fertility (Gómez‐Aparicio, 2009; Bai et al., 2019). In recent years, however, frequent droughts induced by climate change have triggered the widespread withering and death of woody plants, which had severe impacts on ecosystem patterns and processes (Breshears et al., 2009; McDowell and Allen, 2015). For example, woody plant mortality events induced by drought have been reported internationally, i.e., in Alaskan and Amazonian rainforests, Mediterranean Europe, Australia, boreal forests of North America, and semiarid forests of the Southwest United States (Phillips et al., 2009; Williams et al., 2013; McDowell and Allen, 2015). As a consequence of water resource shortages and climate change, the central areas of Inner Mongolia, the northwestern areas of Xinjiang, and the northern areas of Shaanxi within the TNSP have experienced degradation such as canopy withering and even plant death (Yu et al., 2021). Thus, it is very important to understand the drought tolerance and adaptability of replanted shrubs in changing environments to guide revegetation practices.

Revegetation with native shrubs has been one of the most effective ways to control desertification (Zhao et al., 2013; Tian et al., 2019). This is owing to the fact that native shrubs can physiologically adapt to local climates more quickly than non-native species, and they have a strong ability to resist sand burial because the adventitious buds of their branches can give rise to roots and seedlings after sand burial (Luo and Zhao, 2019; Ma et al., 2019). Desert shrubs have extensive geographical range and have evolved a variety of morphological and physiological characteristics and life-history strategies (Xu et al., 2007; De Micco and Aronne, 2012), e.g., desert shrubs vary in crown size, life form, and root system structures (Zhang et al., 2009; Venturas et al., 2016). Additionally, leaf morphology can vary such as the production of smaller-size, split, or degenerated leaves (Zou et al., 2010; Zhang et al., 2016a), which can reduce water consumption in arid habitats (Abd El-Ghani et al., 2017). Correspondingly, the physiological characteristics of desert shrubs also vary with the environmental conditions to adapt to limited water availability (Liu et al., 2021), such as lower water potential at the turgor loss point (Ψ_tlp_) and less xylem hydraulic conductivity (Scholz et al., 2012; Zhou et al., 2013). Additionally, desert shrub species are more resistant to embolism (more negative P_50_) owing to their ability to tolerate very negative water potentials (Lopez et al., 2005). Thus, they have higher xylem hydraulic safety margins that can avoid mortality triggered by short-term drought (Xu et al., 2011; Li et al., 2020). Unquestionably, the variation in morphological and physiological characteristics of desert plants results from life history strategies shaped in long-term adaptation to drought conditions and are the main reason for their survival in harsh desert habitats. However, how desert shrubs physiologically adapt to arid habitats through hydraulic strategies remains unclear.

In recent years, numerous studies have found that various functional traits of plants are coordinated or exhibit trade-offs with each other during physiological adaption to environmental changes (Henry et al., 2019; Rosas et al., 2019). Among these studies, the trade-off between hydraulic efficiency (stem-specific hydraulic conductivity, *K_s_*) and hydraulic safety (the water potential at 50% loss of *K_s_*, P_50_) is the most widely studied (Ocheltree et al., 2016). Woody plants with more negative P_50_ lead to increased tolerance of drought and sustain hydraulic conductivity (Choat et al., 2018). However, many species have low hydraulic efficiency and low hydraulic safety, which might be associated with other traits, such as wood density or leaf-to-sapwood area (Gleason et al., 2016). A trade-off also exists between hydraulic safety and capacitance (De Guzman et al., 2017). Species with high hydraulic capacitance survive during drought even without high safety because capacitance buffers hydraulic failure (Santiago et al., 2018). Additionally, coordination between hydraulic traits and other traits plays an important role in drought response strategies of species (Santiago et al., 2018), e.g., xylem hydraulic conductance coordinated with leaf gas exchange (Rodríguez-Gamir et al., 2021). However, although desert plants have evolved a series of life history traits in response to frequent drought, including critical morphological and physiological characteristics (Xu et al., 2007; De Micco and Aronne, 2012), the role of hydraulics in their whole-plant life history strategies remains unclear. Therefore, identifying the hydraulic strategies of desert shrubs with various life forms and morphologies is important for understanding the drought tolerance and survival of desert shrubs under arid habitats.

In this study, eight desert shrub species that grow in the same arid habitat but differ in life form and morphology were used to study the hydraulic strategies of desert plants mediated by leaf hydraulic traits. Our aims included the following: (1) evaluating the difference in leaf hydraulic traits among species and (2) revealing the trade-off and coordination among leaf hydraulic traits to identify hydraulic strategies.

## Materials and methods

### Study site and species

The study was conducted at the Shapotou Desert Research and Experiment Station, Chinese Academy of Sciences. The station is located at the southeast edge of the Tengger Desert in northern China (37°33′N, 105°02′E). The area is covered by dense and continuous reticulate barchan dunes, and its gravimetric moisture content is about 3%–4%. The mean annual temperature is 9.6°C, the extreme minimum temperature is -25.1°C, and the maximum temperature is 38.1°C. The mean annual precipitation is 186.2 mm. There are about 50 days of precipitation exceeding 0.1 mm, and approximately 80% of the precipitation days were less than 5 mm of precipitation (Zhang et al., 2016b). The average air relative humidity is 40% with a minimum value of 10%. The mean annual wind speed is 2.9 m·s^−1^, mainly northwesterly. The potential evapotranspiration during the growing season (May–September) is 2,300 to 2,500 mm (Zhang et al., 2014).

**Figure 1:**
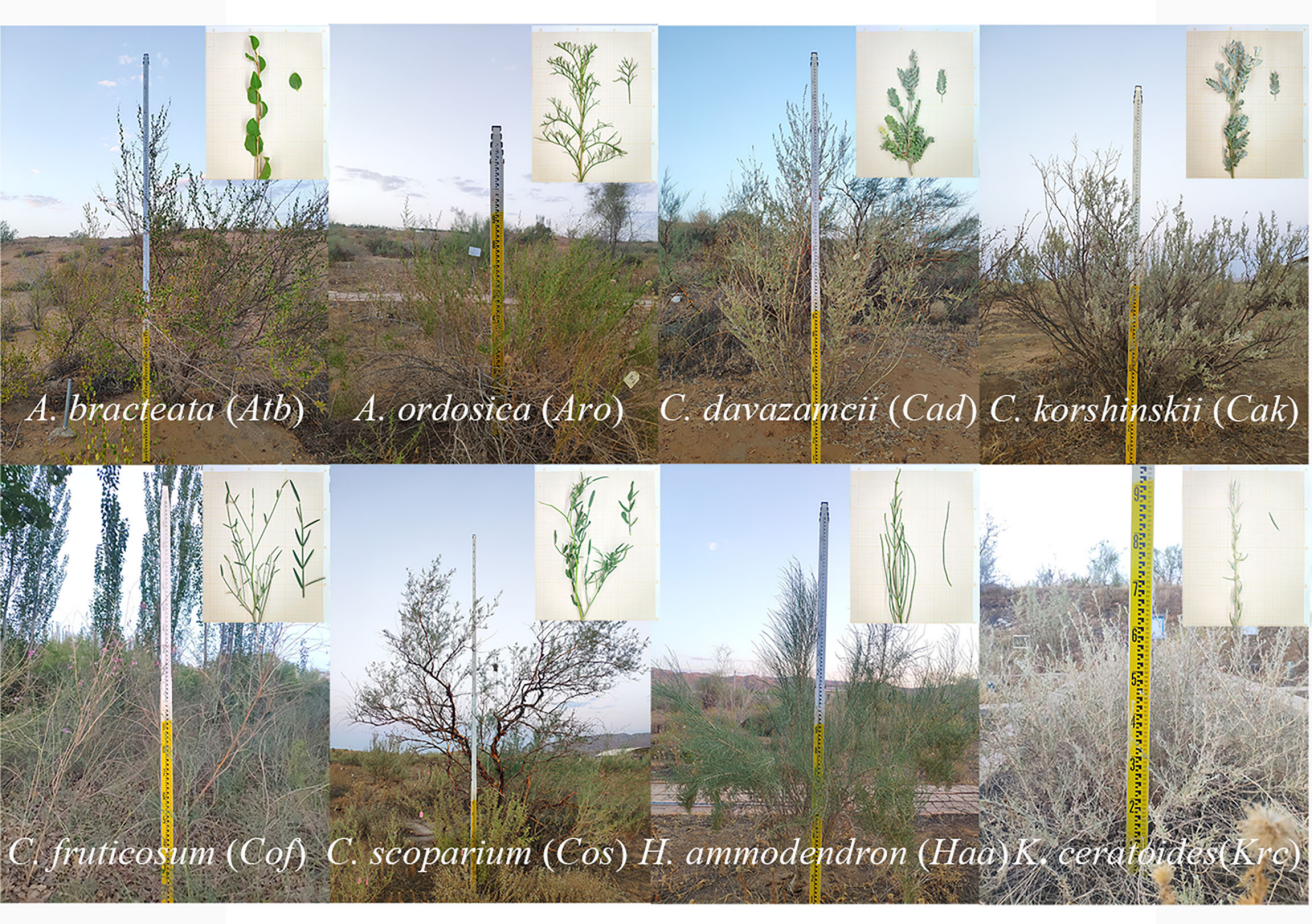
The shrubs and leaf morphology of eight desert shrubs in the southeast edge of the Tengger Desert in northern China.

In 2010, the New Water Balance Experimental Fields (NWBEF; **Supplementary Figure 1**) were constructed by first leveling sand dunes and then erecting sand barriers using a 1 m × 1 m wheat-straw checkerboard pattern; next, 2-year-old seedlings introduced from the sandy areas of northern China were respectively planted at densities of 35 and 70 individuals per 100 m^2^ for shrubs and subshrubs, and each plot area was 600 m^2^. Subsequently, they grew in natural conditions without irrigation. This study was conducted from August to September in 2020, comprising eight species, namely, *Atraphaxis bracteata*, *Artemisia ordosica*, *Caragana davazamcii (synonym: Caragana intermedia)*, *Caragana korshinskii*, *Krascheninnikovia ceratoides (synonym: Ceratoides latens) *, *Haloxylon ammodendron*, *Corethrodendron fruticosum* and *Corethrodendron scoparium*, which were selected from the NWBEF to study hydraulic traits (**Figure 1**). Among them, *C*. *davazamcii*, *C*. *korshinskii*, *C*. *fruticosum*, and *C*. *scoparium* are in the family Fabaceae, whereas *K*. *ceratoides* and *H*. *ammodendron* are in the family Amaranthaceae. The species *A. ordosica* and *A. bracteata* are from the families Compositae and Polygonaceae, respectively. These eight desert shrubs species are widely distributed in northern China (**Supplementary Figure 2**) and showed a larger difference in morphologies among genera (**Figure 1**; **Supplementary Table 1**). Two subshrub species *A. ordosica and K. ceratoides* have shallow root systems (mostly fine roots distributed in the upper 0.4 m of soil) with a broad lateral range to adequately absorb rainwater (Liu et al., 1991; Zhang et al., 2008); however, the other six shrub species have deep root systems (>3-m depth) that collect groundwater (Liu et al., 1991; Zhang et al., 2009; Feng et al., 2022). *A. ordosica* has full split needled leaves, whereas the leaves of *C. davazamcii* and *C. korshinskii* are pinnately compound with three to eight pairs of densely pilose leaflets (Zhang et al., 2016a). *C*. *fruticosum* and *C*. *scoparium* have linear oblong or narrowly lanceolate and small gray-green leaves. However, the leaves of *H. ammodendron* have been degenerated into squamous, and its succulent twigs perform photosynthesis using the C4 pathway (Zou et al., 2010). Moreover, the leaves of *K*. *ceratoides* are small (1–2 cm long), strip-lanceolate, lanceolate, or oblong, whereas *A. bracteate* leaves are leathery and oblong or oval. In this study, five individuals of each species were selected to investigate morphological traits (**Table 1**; **Supplementary Table 2**) and leaf hydraulic traits. During the experiment in August and September, there was no significant difference in soil water contents among different plots, and the precipitation in August and September accounts for 44% of the annual precipitation (**Supplementary Figure 3**).

**Table 1:** Information of the eight desert shrubs species used in this study.

Species	Families	*Abbreviation*	*Symbol*	*H_p_* (m)	*C_w_* (m)	*LA* (cm^2^)
*Atraphaxis bracteata*	Polygonaceae	*Atb*	▼	2.22 ± 0.102 b	2.27 ± 0.125 b	5.25 ± 0.287 a
*Artemisia ordosica*	Compositae	*Aro*	▽	1.02 ± 0.036 de	1.62 ± 0.091 c	1.49 ± 0.112 c
*Caragana davazamcii*	Fabaceae	*Cad*	□	2.27 ± 0.149 b	1.72 ± 0.089 c	2.47 ± 0.117 b
*Caragana korshinskii*	Fabaceae	*Cak*	★	1.59 ± 0.033 c	2.43 ± 0.117 b	2.02 ± 0.085 bc
*Corethrodendron fruticosum*	Fabaceae	*Cof*	▲	1.23 ± 0.088 d	1.05 ± 0.144 d	4.59 ± 0.388 a
*Corethrodendron scoparium*	Fabaceae	*Cos*	○	2.72 ± 0.194 a	3.39 ± 0.218 a	2.51 ± 0.345 b
*Haloxylon ammodendron*	Amaranthaceae	*Haa*	△	1.61 ± 0.113 c	1.47 ± 0.169 cd	2.47 ± 0.129 b
*Krascheninnikovia ceratoides*	Amaranthaceae	*Krc*	◆	0.888 ± 0.052 d	1.36 ± 0.113 cd	0.639 ± 0.055 d

plant height (H_p_, m), crown width (C_w_, m), and leaf area (LA, cm^2^). All values are shown as mean ± SE, and letters indicate the existence of statistical significance among species (one-way ANOVA, n = 5, p < 0.05).

### Water potential measurements

The water potential (Ψ) of sun-exposed leafy shoots was measured using a pressure chamber (1505D-EXP, PMS Instrument Company, Albany, OR, USA) on five individuals per species (three leaves of each individual) before dawn (Ψ_pd_, local time, 5:30–6:30) and at mid-afternoon (Ψ_md_, 13:00–14:00 when the air temperature is the highest, which is considered to correspond to the minimum daily water potential) on 28 August 2020. The daily maximum water potential difference (difference between Ψ_pd_ and Ψ_md_, *Δ*Ψ = Ψ_pd_ - Ψ_md_) was also calculated.

### Leaf hydraulic conductivity and vulnerability curves

For the vulnerability curves (VCs), we determined the leaf hydraulic conductance (*K_l_*, mmol·m^−2^·s^−1^·MPa^−1^) using a timed rehydration method described by Brodribb and Holbrook (2003) and Johnson et al. (2018), which is based on an analogy between rehydrating a leaf and discharging a capacitor as *K_l_* = *C_l_* ln (Ψ_o_/Ψ_f_)/*t*, where *C_l_* is the capacitance of leafy shoots (mmol·m^−2^·MPa^−1^), Ψ_o_ is the leaf water potential prior to partial rehydration (MPa), Ψ_f_ is the leaf water potential after partial rehydration, and *t* is the duration of rehydration (s). Briefly, the collected leafy shoots (10 cm) were placed in containers to rehydrate for at least 4 h and then dried on the bench top for different time periods to reach a range of leaf water potentials under room temperature. Leafy shoots were placed inside black plastic bags containing moist paper towels to allow them to equilibrate in dark conditions for at least 1 h. The Ψ_o_ value of leafy shoots was measured, and then, two adjacent leaves of the same shoots were cut under water and rehydrated for a time period of *t* (ranging from 10 to 60 s) before Ψ_f_ was measured. The VCs were plotted as *K_l_* against Ψ_o_ using 10–20 shoots per species, and three VCs were established for each species based on three individuals. Maximum leaf hydraulic conductance (*K_max_*) was determined by averaging the five highest *K_l_* values per species. The water potential at 50% loss of maximum leaf hydraulic conductance (P_50_, MPa) was determined using the P_50_ value of shoots, which were calculated by fitting a three-parameter sigmoidal regression function of the form Ψ_o_ = a/[1 + e^-k (^*^Kl^* ^-xc)^] to the *K_l_* versus Ψ_o_ data (Blackman et al., 2010; Johnson et al., 2018), where k and xc are constant terms of the equation. Additionally, in order to determine the leaf hydraulic safety margin of embolism occurring (LSM_eo_) and leaves wilting (LSM_lw_), the LSMs at 50% loss of conductivity and at the turgor loss point were calculated as the difference between Ψ_md_ and P_50_ (LSM_eo_ = Ψ_md_ - P_50_, MPa) and the difference between Ψ_md_ and Ψ_tlp_ (LSM_lw_ = Ψ_md_- Ψ_tlp_, MPa), respectively (Ziegler et al., 2019).

### Pressure–volume curves

To establish pressure–volume (P–V) curves, we collected three 10-cm shoots from each shrub and immediately their measured fresh mass (FM, g) before placing them into a container with deionized water at room temperature for 6 h until complete saturation. After reaching full saturation, we removed the shoots from the containers, wiped away excess surface water, and measured the saturation mass (SM, g). We weighed the shoots using an analytical balance (AR2140, 1/10 000 accuracy; Ohaus International Trade (Shanghai) Co., Ltd., Shanghai, China) before measuring the balancing pressure. We then immediately placed shoots in the pressure chamber (1505D-EXP) to measure the initial balancing pressure of each sample. Then, chamber pressure was successively raised by 0.1- to 0.15-MPa increments at a speed of about 0.025 MPa s^-1^ and kept for 5 min under each target pressure at room temperature (Huo et al., 2021). The sample was reweighed after completing each measurement. The above operation was repeated more than 10 times until the maximum equilibrium pressure reached 4~5 MPa. Then, we measured the dry mass (DM, g) of samples after drying them in an oven at 75°C for 48 h.

P–V curves were established based on the relative water deficit (RWD; %) versus the reciprocal balance pressure (1/P) according to Nardini et al. (2013). All data points were connected to form a curve, and the entire curve was respectively fitted to a power function (1/P = *α*RWD*^β^*) and a linear function (1/P = *a* + *b*RWD) with loss of turgor pressure as the transition point between the two functions (He et al., 2007), where *α*, *β*, *a*, and *b* are the equation coefficients. The traits, including osmotic potential at saturation (Ψ_π, sat_, MPa), water potential at the turgor loss point (Ψ_tlp_, MPa), relative water content at the turgor loss point (RWC_tlp_, %), symplastic water content (SWC), and bulk tissue modulus of elasticity (*ϵ*, MPa), were estimated from the P–V curve (Tyree and Hammel, 1972; Nardini et al., 2013). Additionally, the sensitivity coefficients of water potential change before or after the turgor loss point, −1/*β* and −1/*b*, reflect the change in −1/Ψ for a unit change in the RWD of a species before and after the turgor loss point. We calculated these sensitivity coefficients according to Huo et al. (2021). The hydraulic capacitance (*C*, mmol · m^−2^ · MPa^−1^) values of both pre-turgor loss and post-turgor loss (*C_pre-tlp_* and *C_pos-tlp_*) were estimated from the P–V curves according to Brodribb and Holbrook (2003). The ratios of DM to LA and SM to DM were determined for each species and used in the following equation to calculate the leaf area normalized absolute capacitance: *C_leaf_* = *Δ*RWC/*Δ*Ψ × (DM/LA) × (SM/DM)/M. Here, *Δ*RWC and *Δ*Ψ are the difference in leaf relative water content (%) and water potential (MPa) between before and after turgor loss points, respectively, DM is the leaf dry mass (g), LA is the projected leaf area (m^2^), SM is the mass of leaf water (g) at 100% RWC, and M is the molar mass of water (g·mol^-1^). The total hydraulic capacitance (*C_total_*) is the sum of *C_pre-tlp_* and *C_pos-tlp_*.

### Data analysis

One-way analysis of variance (ANOVA) followed by a Tukey *post-hoc* test was used to determine significant differences in leaf hydraulic functional traits among species and genera. Phylogenetic trees of the eight species in this study were built from the mega-tree ‘GBOTB.extended.tre’ using the R package ‘V.PhyloMaker’ (Jin and Qian, 2019). Principal component analysis (PCA) was performed to evaluate the multiple relationships among functional traits and species. The species scores comprising the first and second components of the PCA were extracted. The relationships among leaf hydraulic functional traits and drought traits were evaluated by linear regression analyses. Abbreviations of various functional traits are presented in **Table 2**. All statistical analyses, figure plotting, and curve fitting were performed using Origin version 2019b (OriginLab Corp., Northampton, MA, USA).

**Table 2:** Abbreviations and units of functional traits in this study.

Functional trait	Abbreviation	Units
Plant height	*H_p_*	m
Crown width	*C_w_*	m
Leaf area	*LA*	cm^2^
Leaf length	*L_l_*	cm
Leaf width	*W_l_*	cm
Leaf water potential at predawn	Ψ_pd_	MPa
Leaf water potential at midday	Ψ_md_	MPa
Diurnal leaf water potential change (Ψ_pd_ - Ψ_md_)	*Δ*Ψ	MPa
Maximum leaf hydraulic conductance	*K_max_*	mmol·m^−2^·s^−1^·MPa^−1^
Water potential at 50% loss of maximum leaf hydraulic conductance	P_50_	MPa
Leaf hydraulic safety margins of embolism occurring	LSM_eo_	MPa
Leaf hydraulic safety margins of wilting	LSM_lw_	MPa
Osmotic potential at saturation	Ψ_π, sat_	MPa
Water potential at turgor loss	Ψ_tlp_	MPa
Relative water content at turgor loss	*RWC_tlp_*	%
Bulk tissue modulus of elasticity	*ϵ*	MPa
Water sensitivity coefficients before turgor loss point	-1/*β*	MPa·%^-1^
Water sensitivity coefficients after turgor loss point	−1/*b*	MPa·%^-1^
Leaf hydraulic capacitance of pre-turgor loss point	*C_pre-tlp_*	mol·m^-2^·MPa^-1^
Leaf hydraulic capacitance of post-turgor loss point	*C_pos-tlp_*	mol·m^-2^·MPa^-1^
Total leaf hydraulic capacitance	*C_total_*	mol·m^-2^·MPa^-1^

## Results

### Leaf water potential, hydraulic efficiency and safety, and hydraulic safety margins

**Figure 2:**
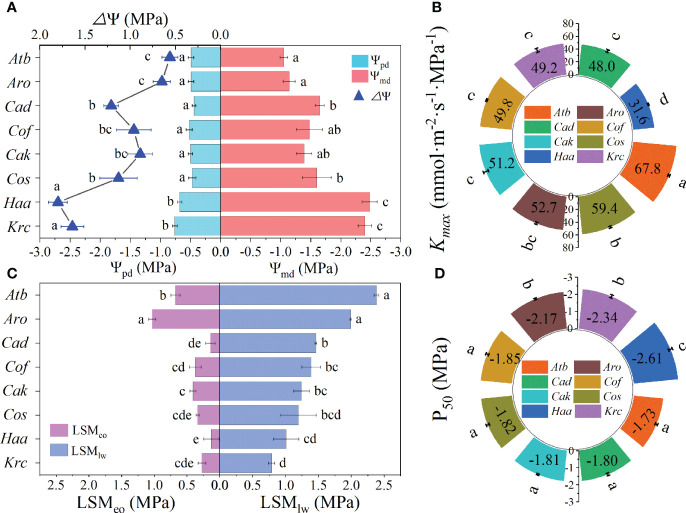
Leaf hydraulic functional traits of desert shrubs. **(A)** Leaf water potential at predawn (Ψ_pd_) and at midday (Ψ_md_), and diurnal leaf water potential change (△Ψ). **(B)** Maximum leaf hydraulic conductance (*K*_max_). **(C)** Leaf hydraulic safety margins of embolism occurring (LSM_eo_) of leaves wilting (LSMl_w_). **(D)** Water potential at 50% loss of maximum leaf hydraulic conductance (P50). Abbreviations of species name and functional traits are shown in **Tables 1**, **2**. All values are shown as mean ± SE, and different letters indicate the existence of statistical significance among species (one-way ANOVA, n = 5, *p* < 0.05).

For the eight species of desert shrubs, Ψ_pd_ value of leaves were relatively stable at -0.50 MPa (**Figure 2A**) except for *H*. *ammodendron* and *K*. *ceratoides* (which were significantly more negative than those of the other six species). However, Ψ_md_ varied from -1.05 in *A*. *bracteata* to -2.48 MPa in *H*. *ammodendron* and was more negative than Ψ_pd_. Among the Ψ_md_, those of *K*. *ceratoides* and *H*. *ammodendron* were significantly lower than those of other species, whereas *A*. *ordosica* and *A*. *bracteata* had significantly higher Ψ_md_ than the other four species (**Figure 2A**). The *Δ*Ψ values for *K*. *ceratoides* and *H*. *ammodendron* (1.80 and 1.64 MPa) were larger than those of the other species (**Figure 2A**). The larger *K_max_* were respectively found in *A*. *bracteata* and *C. scoparium*, which were 67.8 and 59.4 mmol·m^-2^·s^-1^·MPa^-1^, and *H*. *ammodendron*’s *K_max_* (31.6 mmol·m^-2^·s^-1^·MPa^-1^) was significantly lower than those of the other species (**Figure 2B**). Moreover, there was no significant difference among the other five species. The P_50_ estimated from VCs (**Supplementary Figure 4**) of *H*. *ammodendron*, *K*. *ceratoides*, and *A*. *ordosica* were -2.61, -2.34, and -2.17 MPa, respectively, which were more negative than those of the other species (**Figure 2D**). The other five species’ P_50_ had no obvious differences among them (**Figure 2D**). LSM_eo_ and LSM_lw_ values of *A*. *bracteata* and *A*. *ordosica* were highest (**Figure 2C**). However, *H*. *ammodendron*’s LSM_eo_ was significantly lower than those of the other five species, which had LSM_eo_ and LSM_lw_ that were not significantly different (**Figure 2C**). Additionally, other differences in Ψ_pd_, Ψ_md_, *Δ*Ψ, *K_max_*, and P_50_ were also observed among the different genera (**Supplementary Figure 5**).

### The traits of shrubs from pressure–volume curves

**Figure 3:**
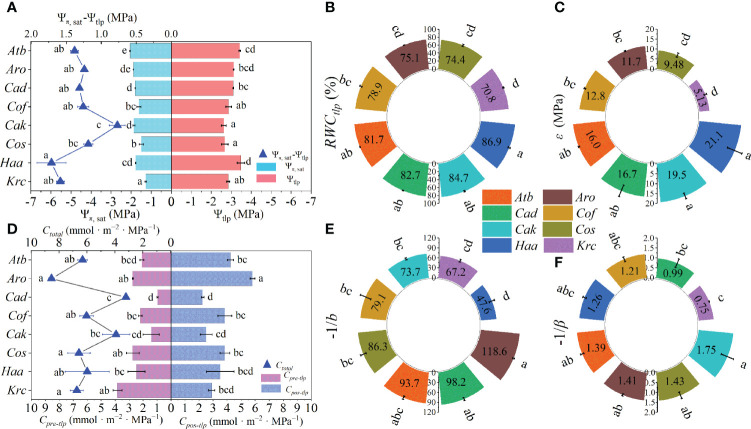
The traits of eight desert shrubs estimated from P–V curves. **(A)** Osmotic potential at saturation (Ψ_π_, _sat_), water potential at turgor loss (Ψ_tlp_) and the difference value between them. **(B)** Relative water content at turgor loss (RWC_tlp_). **(C)** Bulk tissue modulus of elasticity (ε). **(D)** Leaf hydraulic capacitance of pre-turgor loss point (C_pre-tlp_) and post-turgor loss point (C_pos-tlp_), and total leaf hydraulic capacitance (C_total_). **(E, F)** Water sensitivity coefficients before turgor loss point (−1/b) and after turgor loss point (−1*/b*). Abbreviations of species name and functional traits are shown in **Tables 1**, **2**. All values are shown as mean ± SE, and different letters indicate the existence of statistical significance among species (one-way ANOVA, n = 5, *p* < 0.05).

The traits, including Ψ_π, sat_, Ψ_tlp_, and *ϵ*, were estimated from the P–V curves (**Supplementary Figure 6**). More negative Ψ_tlp_ than Ψ_π, sat_ was observed among all shrubs (**Figure 3A**). More negative Ψ_π, sat_ was found in *A*. *bracteata*, *A*. *ordosica*, *C*. *davazamcii*, and *C*. *korshinskii*, whereas the Ψ_π, sat_ of *K*. *ceratoides* and *C*. *scoparium* were larger than those of the other species (**Figure 3A**). The largest Ψ_tlp_ were observed in *C*. *scoparium* and *C*. *korshinskii*, but *H*. *ammodendron* had the smallest Ψ_tlp_ (**Figure 3A**). The difference between Ψ_π, sat_ and Ψ_tlp_ (i.e., Ψ_π, sat_-Ψ_tlp_) of *C*. *korshinskii* was significantly lower than those of others species, and this difference was obviously larger in *H*. *ammodendron* than in *C*. *scoparium*. However, the five other shrub species showed no significant difference in this value (the blue triangle in **Figure 3A**). The largest RWC_tlp_ and smallest *ϵ* values were all found in *H*. *ammodendron* and *K*. *ceratoides* (**Figures 3B, C**). Regarding hydraulic capacitance, the minimum *C_pre-tlp_*, *C_pos-tlp_*, and *C_total_* values were found in *C*. *davazamcii*, whereas the *C_pos-tlp_* and *C_total_* of *A*. *ordosica* were significantly larger than those of the other species (**Figure 3D**). Meanwhile, the *C_pre-tlp_* of the other seven shrubs except for *K*. *ceratoides* were less than their respective *C_pos-tlp_* values (**Figure 3D**). Moreover, higher -1/*b* were found in *A*. *ordosica* than in other shrubs, except for *A*. *bracteate* and *C*. *davazamcii*, whereas the -1/*b* value of *H*. *ammodendron* was obviously lower than those of the other species, apart from *K*. *ceratoides* (**Figure 3E**). The -1/*β* of *C*. *korshinskii* was only significantly larger than those of *C*. *davazamcii* and *K*. *ceratoides*, but there was no significant difference among the other species (**Figure 3F**). Additionally, differences in Ψ_π, sat_, Ψ_tlp_, *C_pre-tlp_*, *C_pos-tlp_*, and *C_total_* were also observed among genera (**Supplementary Figure 5**).

### Relationship among leaf hydraulic functional traits

**Figure 4:**
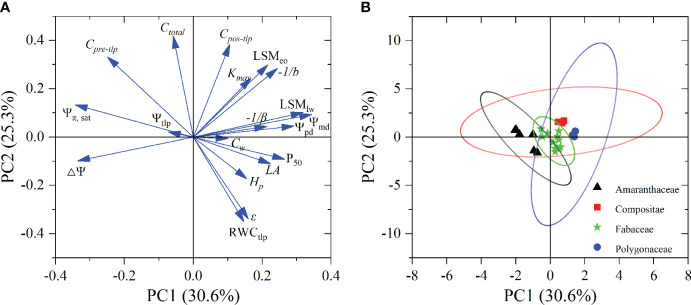
Factor loadings **(A)** and species scores **(B)** of PCA for 19 functional traits in eight desert shrubs species. Abbreviations of species name and functional traits are shown in **Tables 1**, **2**. The ovals with different colored represent species belonging to different families in **(B)**. Ellipses represent 90% confidence intervals for different families.

Principal component analysis (PCA) results based on the 19 functional traits of the eight desert shrub species showed that the first and second the components accounted for 30.6% and 25.3% of the total variance, respectively (**Figure 4**; **Supplementary Figure 7**). The traits related to water status (i.e., Ψ_pd_ and Ψ_md_) and sensitivity to water potential changes (-1/*b* and -1/*β*) showed positive loading on the first PCA component, whereas only Ψ_π, sat_ and *△*Ψ had negative loading on the first component. The second component was positively loaded by hydraulic capacitance (i.e., *C_pre-tlp_*, *C_pos-tlp_*, and *C_total_*) and leaf hydraulic safety margin (LSM_eo_), whereas RWC_tlp_ and *ϵ* had negative loadings on the second component (**Figure 4A**; **Supplementary Table 3**). However, morphological traits (*H_p_* and *C_w_*), leaf hydraulic efficiency (*K_max_*), and leaf hydraulic safety (P_50_) showed positive loading on the third component (**Supplementary Table 3**). Species appear to be separated among the PCA component by family (**Figure 4B**; **Supplementary Figure 8**). *Artemisia* and *Atraphaxis* were showed positively loading on first component, whereas Amaranthaceae family species tended to have negative first component values. However, Fabaceae family species had both negative and positive second component values (**Figure 4B**; **Supplementary Figure 8**).

**Figure 5:**
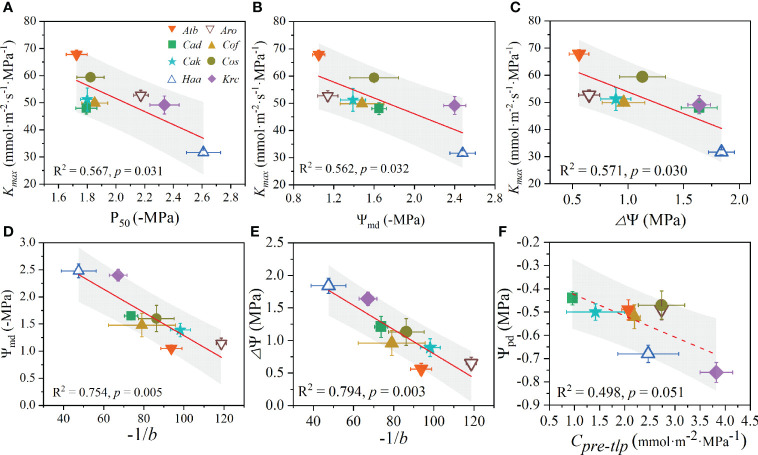
Trade-offs among leaf hydraulic functional traits of desert shrubs. The relationships between Kmax with P_50_ **(A)**, Ψ_md_ **(B)** and △Ψ **(C)**. The relationships between −1*/b* with Ψ_md_ **(D)** and △Ψ **(E)**. The relationship between Ψ_pd_ and C_pre-tlp_ **(F)**. All values are shown as mean ± SE. Abbreviations of species name and functional traits are shown in **Tables 1**, **2**. The coefficients of determination (R^2^) and significance levels (*p*) of linear regression are shown. Shades of gray represent 90% confidence intervals.

**Figure 6:**
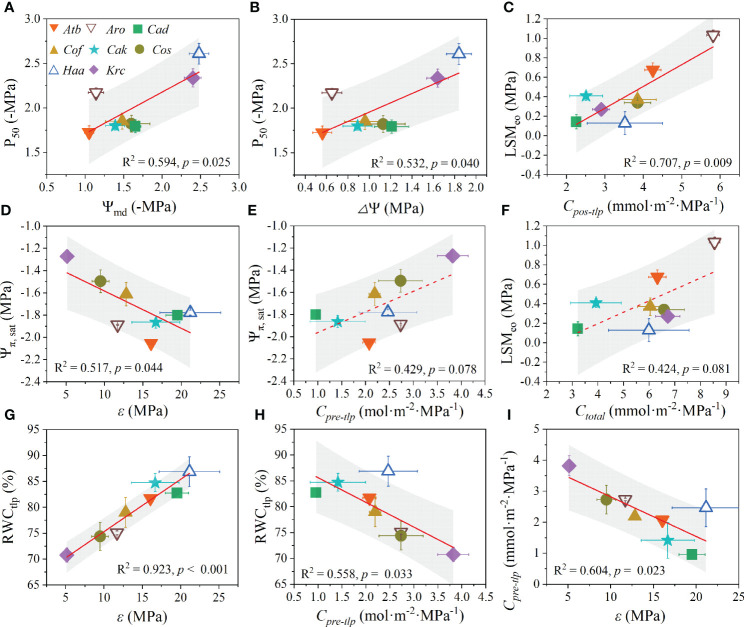
Coordination among leaf hydraulic functional traits of desert shrubs. The relationships between P_50_ with Ψ_md_ **(A)** and △Ψ **(B)**. The relationships between LSM_eo_ with C_pos-tlp_ **(C)** and C_total_ **(F)**. The relationships between Ψ_π_, _sat_ with ε **(D)** and C_pre-tlp_ **(E)**. The relationships between RWCtlp with *ε* **(G)** and C_pre-tlp_ **(H)**. The relationship between *ε* and C_pre-tlp_ **(I)**. All values are shown as mean ± SE. Abbreviations of species name and functional traits are shown in **Tables 1**, **2**. The coefficients of determination (R^2^) and significance levels (*p*) of linear regression are shown. Shades of grey represent 90% confidence intervals.Tables.

In this study, *K_max_* showed a strong significantly negative relationship with P_50_ (**Figure 5A**), Ψ_md_ (**Figure 5B**), and *Δ*Ψ (**Figure 5C**). Additionally, the sensitivity coefficient -1/*b* was significantly negatively related to Ψ_md_ (**Figure 5D**) and *Δ*Ψ (**Figure 5E**). However, a marginally negative correlation was observed between Ψ_pd_ and *C_pre-tlp_* (**Figure 5F**). Importantly, a positive relationship was found between P_50_ with both Ψ_md_ (**Figure 6A**) and *Δ*Ψ (**Figure 6B**). Likewise, LSM_eo_ exhibited a significant positive relationship with *C_pos-tlp_* (**Figure 6C**), but a marginally positive relationship was observed between LSM_eo_ and *C_total_* (**Figure 6F**). The Ψ_π, sat_ showed a significant negative correlation with *ϵ* (**Figure 6D**) and a marginally positively relationship with *C_pre-tlp_* (**Figure 6E**). Conversely, RWC_tlp_ showed a significant positive relationship with *ϵ* (**Figure 6G**) and a significant negative relationship with *C_pre-tlp_* (**Figure 6H**). In addition, *C_pre-tlp_* showed a significantly negative correlation with *ϵ* (**Figure 6I**). Unexpectedly, the morphological traits (*H_p_*, *C_w_*, *LA*, *L_l_*, and *W_l_*) exhibited no significant relationships with leaf hydraulic functional traits (*K_max_*, P_50_, LSM_eo_, *Δ*Ψ, Ψ_π, sat_, RWC_tlp_, *ϵ*, and *C_pre-tlp_*; **Supplementary Figure 9** and 10), except for one; the linear relationship between *W_l_* and *Δ*Ψ was significantly negative (R^2^ = 0.707, *p* = 0.009).

## Discussion

### Differences in leaf functional traits among desert shrubs

Numerous studies have shown large interspecies differences in hydraulic functional traits (Chen et al., 2009; Fan et al., 2011; Borghetti et al., 2020). Our study found more negative Ψ_pd_, Ψ_md_, and P_50_ values; larger *Δ*Ψ; and smaller *K_max_* in *H. ammodendron* and *K. ceratoides* than in *A. ordosica* and *A. bracteate*. These results indicated that shrubs in the Amaranthaceae family with more negative Ψ_md_ and P_50_ have strong tolerance and resistance against embolism, whereas shrubs in the Polygonaceae and Compositae families are more vulnerable to embolism due to their greater hydraulic conductance ensuring small water potential changes and less negative P_50_. Although the root systems play important roles in drought tolerance, the root distribution of *A. ordosica* (shallow root system) was not related to water potential changes and hydraulic safety in the present study, as was deep-rooted shrubs, which may be more related to other traits, e.g., morphological traits. For example, *A. ordosica* with less negative Ψ_pd_, Ψ_md_, and P_50_ may be attributable to its full split needled and semi-succulent leaves but irrespective of root distribution (**Supplementary Table 1**; Liu et al., 1991; Zhang et al., 2008), highlighting that plant morphology can underlie differences in physiology (Possen et al., 2014). The leaf hydraulic safety margin could better explain mortality under continuous or severe droughts than P_50_ or other traits alone (Anderegg et al., 2016). Most angiosperm species with narrow hydraulic safety margin values (<1 MPa) suggest that species are highly vulnerable to increases in the frequency of droughts (Choat et al., 2012; Anderegg et al., 2016). Similarly, our results found narrow LSM_eo_ (<1 MPa) among desert shrubs; however, the desert shrubs had wider LSM_lw_ (Ψ_md_ - Ψ_tlp_; >1 MPa) than LSM_eo_. As Ψ_tlp_ is usually recognized as the key trait quantifying leaf wilting and plant drought tolerance most directly (Bartlett et al., 2012b), LSM_lw_ shows the hydraulic safety margin of leaf wilting in this study. Therefore, our results suggested that desert shrubs were prone to embolism, but not quick to wilt during drought. This seems to suggest that embolism is not fatal to desert shrubs and that proper embolization of desert shrubs can indeed reduce water loss to support their basic survival. Finally, shrubs in the Amaranthaceae family have more narrow LSM_eo_ and LSM_lw_ relative to Polygonaceae and Compositae family shrubs, indicating that Amaranthaceae family shrubs are more prone to wilting than the latter species owing to their narrow hydraulic safety margins caused by larger daily water potential differences.

Recently, some studies have shown that Ψ_π, sat_ and Ψ_tlp_ are key characteristics for predicting drought tolerance (Bartlett et al., 2012a). In the present study, we found more negative Ψ_tlp_, Ψ_π, sat_ and largest Ψ_π, sat_ - Ψ_tlp_ in *H. ammodendron* relative to those in *C. korshinskii* and *C. scoparium*, which suggests that *H. ammodendron* has greater drought tolerance than the latter species because plants with more negative Ψ_tlp_ and Ψ_md_ maintain stomatal and hydraulic conductance (Mitchell et al., 2008; Bartlett et al., 2012b) or because the leaves of *H. ammodendron* are degenerate to limit water loss (Zou et al., 2010). Meanwhile, the maximum and minimum RWC_tlp_ and *ϵ* values were observed in *H. ammodendron* and *K. ceratoides*, showing that *H. ammodendron* with lower cell wall elasticity is prone to loss of cellular water, whereas *K. ceratoides* sacrifices cell wall elasticity to maintain tissue water content (Kozlowski and Pallardy, 2002; Bartlett et al., 2012b). However, RWC_tlp_ and *ϵ* did not significantly differ among the other shrub species. The larger hydraulic capacitance (i.e., *C_pre-tlp_*, *C_pos-tlp_*, and *C_total_*) of *A. ordosica* suggested that it has a strong water storage capacity and larger buffering effect during droughts (Meinzer et al., 2009; Huo et al., 2021), whereas the lower hydraulic capacitance of *C. davazamcii* and *C. korshinskii* makes them more sensitive to water deficit, perhaps owing to their greater water potential change and less negative P_50_. In addition, the water sensitivity coefficient (-1/*β*) was larger in *C. korshinskii*, also indicating that it is more sensitive to water deficit relative to other species. Overall, the significant difference in Ψ_tlp_, Ψ_π, sat_ RWC_tlp_, *ϵ*, *C_pre-tlp_*, *C_pos-tlp_*, and -1/*β* observed among different species suggested that change in these traits reflects differences in drought tolerance responses of species to water deficit (Touchette et al., 2007).

Taken together, there were significant differences in diurnal leaf water potential change, leaf hydraulic efficiency and safety, hydraulic safety margin, water potential and relative water content at the turgor loss point, and hydraulic capacitance among species and genera; however, these hydraulic functional traits among species did not show convergence under the same environment. The above results suggested that leaf hydraulic functional traits were more strongly associated with the species, rather than exhibiting convergence in the same environment where they live together. Overall, the difference in leaf hydraulic functional traits among shrubs species also provided important hydraulic-related insights for native shrubs as a priority species in desert vegetation restoration and reconstruction.

### Trade-off among leaf hydraulic functional traits in desert shrubs

In the past decade, the trade-off between hydraulic safety and efficiency has been widely investigated (Nardini and Luglio, 2014; Bucci et al., 2019). Some studies across woody angiosperms and gymnosperms globally have revealed weak trade-offs between *K_max_* and P_50_ (Gleason et al., 2016; Yan et al., 2020; Liu et al., 2021). However, the evidence of a significant correlation between *K_max_* and P_50_ in this study indicated that the hydraulic safety and efficiency of desert shrubs exhibit a strong trade-off (R^2^ = 0.567, *p* = 0.031). For example, *H. ammodendron* has a low efficiency (lower *K_max_*) but strong resistance to embolism (i.e., more negative P_50_), whereas *A. bracteata* exhibits the opposite trend, which is likely owing to their leaf and canopy structure characteristics (e.g., large crown size and leaf degeneration in *H. ammodendron*, but larger leaf area and leathery leaves in *A. bracteata*) or differences in xylem anatomy, which merits further study. Our results are consistent with previous investigations that suggest strong trade-offs between hydraulic safety and efficiency within numerous single sites (Martınez-Vilalta et al., 2002; Pratt et al., 2007) and among particular taxa (Hacke et al., 2007; Yao et al., 2021). The relationships among traits at the global scale do not necessarily manifest similarly at specific sites or in a specific region—traits that appear closely coordinated at certain scales may have different sensitivities to scale-dependent drivers of variation (Messier et al., 2017), which may be attributed to species evolving different life history traits (physiological and morphological traits) in different environments (Xu et al., 2007; Gupta et al., 2020).

PCA indicated the diurnal leaf water potential change (*Δ*Ψ) and leaf hydraulic efficiency (*K_max_*) show loading with opposite signs for the first component. This result implies that trade-offs exist between diurnal leaf water potential change and hydraulic efficiency. As the minimal leaf water potential (typically Ψ_md_) is the critical point for maintaining normal physiological metabolism and *Δ*Ψ acts as an indicator of drought tolerance (Bhaskar and Ackerly, 2006), a strong negative correlation between *K_max_* and Ψ_md_ indicated a hydraulic trade-off between hydraulic efficiency and the minimal leaf water potential. This trade-off indicated that less drought-tolerant shrubs (i.e., with less negative Ψ_md_ and P_50_, and small *Δ*Ψ) have higher hydraulic efficiency, like *A. bracteate*. This may be because *A. bracteate* with a particularly deep root system (Feng et al., 2022) can maintain higher xylem hydraulic efficiency through absorbing deep soil moisture or has a wide leaf hydraulic safety margin of embolism occurring (larger LSMeo) to avoid massive embolism formation. Additionally, the negative relationship between -*1/b* and both Ψ_md_ and *Δ*Ψ showed trade-offs, suggesting that shrubs with small diurnal leaf water potential change were more sensitive to drops in water potential after tissue turgor loss (Huo et al., 2021).

Overall, our study indicated that hydraulic efficiency (*K_max_*) exhibits a trade-off with both hydraulic safety (P_50_) and the minimal leaf water potential and water potential changes (Ψ_md_ and *Δ*Ψ), and the trade-off also existed between -*1/b* with Ψ_md_ and *Δ*Ψ. This means that desert shrub species with small diurnal leaf water potential change, less negative minimal leaf water potential, and low hydraulic safety were more sensitive to embolism and water deficit.

### Coordination among leaf hydraulic functional traits in desert shrubs

Numerous studies have suggested that there is clear coordination among hydraulic traits (Pivovaroff et al., 2018). For instance, species with high resistance to embolism usually share the following characteristics: higher wood density, more negative leaf Ψ_tlp_, and larger capacitance (Santiago et al., 2018). In the present study, the diurnal leaf water potential change (*Δ*Ψ) and hydraulic safety (P_50_) had both positive and negative loadings on the principal component. Moreover, we found positive correlations between P_50_ with both *Δ*Ψ and Ψ_md_, indicating that leaf hydraulic safety coordinated with the water potential changes and the minimal leaf water potential. That is, desert shrubs with more negative Ψ_md_ and larger *Δ*Ψ were more resistant to embolism, e.g., *H. ammodendron*. Moreover, the shrubs with lower *Δ*Ψ and Ψ_md_ had more negative P_50_ and higher *K_max_*, suggesting that the shrubs with smaller minimal leaf water potential and higher leaf hydraulic safety have lower leaf hydraulic efficiency, which was consistent with the results of previous studies (Nardini and Luglio, 2014; Yan et al., 2020; Liu et al., 2021). The minimal leaf water potential was coordinated with leaf hydraulic safety, whereas it exhibited a trade-off with leaf hydraulic efficiency; therefore, minimal leaf water potential seems to act as a mediator in the trade-off with leaf hydraulic efficiency and in coordination with leaf hydraulic safety. Additionally, the coordination between −1/*b* with Ψ_md_ and *Δ*Ψ suggested that desert shrubs with less negative Ψ_md_ and smaller *Δ*Ψ were more sensitive to drop in water potential after losing turgor pressure (Huo et al., 2021). Since Ψ_md_ is easier to measure than leaf hydraulic safety and reflects the maximum water deficit that species must tolerate to maintain physiological activity (Bhaskar and Ackerly, 2006), we highlight that Ψ_md_ could act as a convenient trait to preliminarily determine or predict leaf hydraulic conductivity and embolism resistance in shrub species.

Hydraulic safety margins are usually used to describe the degree of conservatism in a plant’s hydraulic strategies (Johnson et al., 2012). In our study, the hydraulic capacitance (i.e., *C_pre-tlp_*, *C_pos-tlp_*, and *C_total_*) and leaf hydraulic safety margin (LSM_eo_) had loadings with the same sign for the second component of the PCA. Meanwhile, a significant positive or marginal correlation between LSM_eo_ with *C_pos-tlp_* and *C_total_* indicated that there is coordination between hydraulic safety margins and hydraulic capacitance. That is, desert shrubs with larger hydraulic capacitance possess larger hydraulic safety margins, which may be because the buffering effect of hydraulic capacitance mitigates the drought-induced risks of reducing water potential (Ding et al., 2021). The significant coordination between LSM_eo_ and -1/*b* suggested that desert shrubs with larger hydraulic safety margins to embolism are more sensitive to water deficit after losing turgor pressure. Additionally, the coordination in hydraulic capacitance of the pre-turgor loss point (*C_pre-tlp_*) with other traits (i.e., Ψ_π, sat_, RWC_tlp_, and Ψ_pd_) revealed that hydraulic capacitance plays an important buffering role in the drought tolerance of desert shrubs (Vergeynst et al., 2015). The bulk tissue modulus of elasticity (*ϵ*) had a trade-off with Ψ_π, sat_ and *C_pre-tlp_* but was coordinated with RWC_tlp_, indicating that desert shrubs with large cell wall elasticity (low *ϵ*) have large hydraulic capacitance and saturated osmotic potential and lose less water at the point of turgor loss (Bartlett et al., 2014; Al-Yasi et al., 2020).

Taken together, the minimal leaf water potential could preliminarily determine or predict leaf hydraulic conductivity and resistance to embolism in desert shrubs, because it is coordinated with leaf hydraulic safety and exhibits a trade-off with leaf hydraulic efficiency. Moreover, the coordination among leaf hydraulic capacitance with other traits (i.e., relative water content at the turgor loss point, osmotic potential at saturation, and bulk tissue modulus of elasticity) plays an important and indispensable role in the survival of shrubs in arid habitats. Critically, the coordination among leaf hydraulic functional traits provides an important strategy for the survival of desert shrubs.

## Conclusion

In this study, the difference in the leaf functional traits and hydraulic strategies of eight desert shrub species were analyzed. Significant differences in leaf functional traits (i.e., diurnal leaf water potential change, hydraulic safety margin, leaf hydraulic efficiency and safety, water potential and relative water content at the turgor loss point, and hydraulic capacitance) were found to exist among species and genera, indicating that leaf hydraulic functional traits were more strongly associated with the species even when living in the same environment. Additionally, our results suggested that the minimal leaf water potential of desert shrubs was strongly coordinated with hydraulic safety and traded off with hydraulic efficiency; therefore, minimal leaf water potential may be used to preliminarily determine or predict leaf hydraulic conductivity and the resistance to embolism of desert shrubs. Leaf hydraulic capacitance was also coordinated with leaf hydraulic safety margins to embolism and other traits, suggesting that hydraulic capacitance plays an important buffering role in the drought tolerance of desert shrubs. In short, our study provides critical insight into hydraulic trade-off and coordination strategies for native shrubs as a priority species for desert vegetation restoration and reconstruction.

## Data availability statement

The original contributions presented in the study are included in the article/**Supplementary Material**. Further inquiries can be directed to the corresponding authors.

## Author contributions

ZZ and JH conceived the research and designed experiments. JH analyzed the data and wrote the manuscript. JH, YS, JC, HZ, and LF performed the experiments. ZZ and YZ revised the manuscript. All the authors read and approved the submission of the manuscript.

## Funding

This work was supported by the Major Science and Technology Projects of Inner Mongolia Autonomous Region (2021ZD0008-3-2), the National Natural Science Foundation of China (Grant Nos. 31971529 and 32171630), the Chinese Academy of Sciences “Light of West China” Program and China Postdoctoral Science Foundation (2021M703465).

## Acknowledgments

We greatly appreciate reviewers for their valuable and insightful comments on this manuscript. We would like to thank Dr. Larry Bowman at Yale University for his assistance with English language and grammatical editing.

## Conflict of interest

The authors declare that the research was conducted in the absence of any commercial or financial relationships that could be construed as a potential conflict of interest.

## Publisher’s note

All claims expressed in this article are solely those of the authors and do not necessarily represent those of their affiliated organizations, or those of the publisher, the editors and the reviewers. Any product that may be evaluated in this article, or claim that may be made by its manufacturer, is not guaranteed or endorsed by the publisher.
