# Supplementary material for: Hydraulic trade-off and coordination strategies mediated by leaf functional traits of desert shrubs
Source: Front Plant Sci. 2022 Oct 31;13:938758. doi: 10.3389/fpls.2022.938758 (PMC9662791; doi:10.3389/fpls.2022.938758)
Supplement: Supplementary file 1 [file DataSheet_1.docx]

Supplementary materials

**1 Supplementary Figures and Tables**

**1.1 Supplementary Figures**


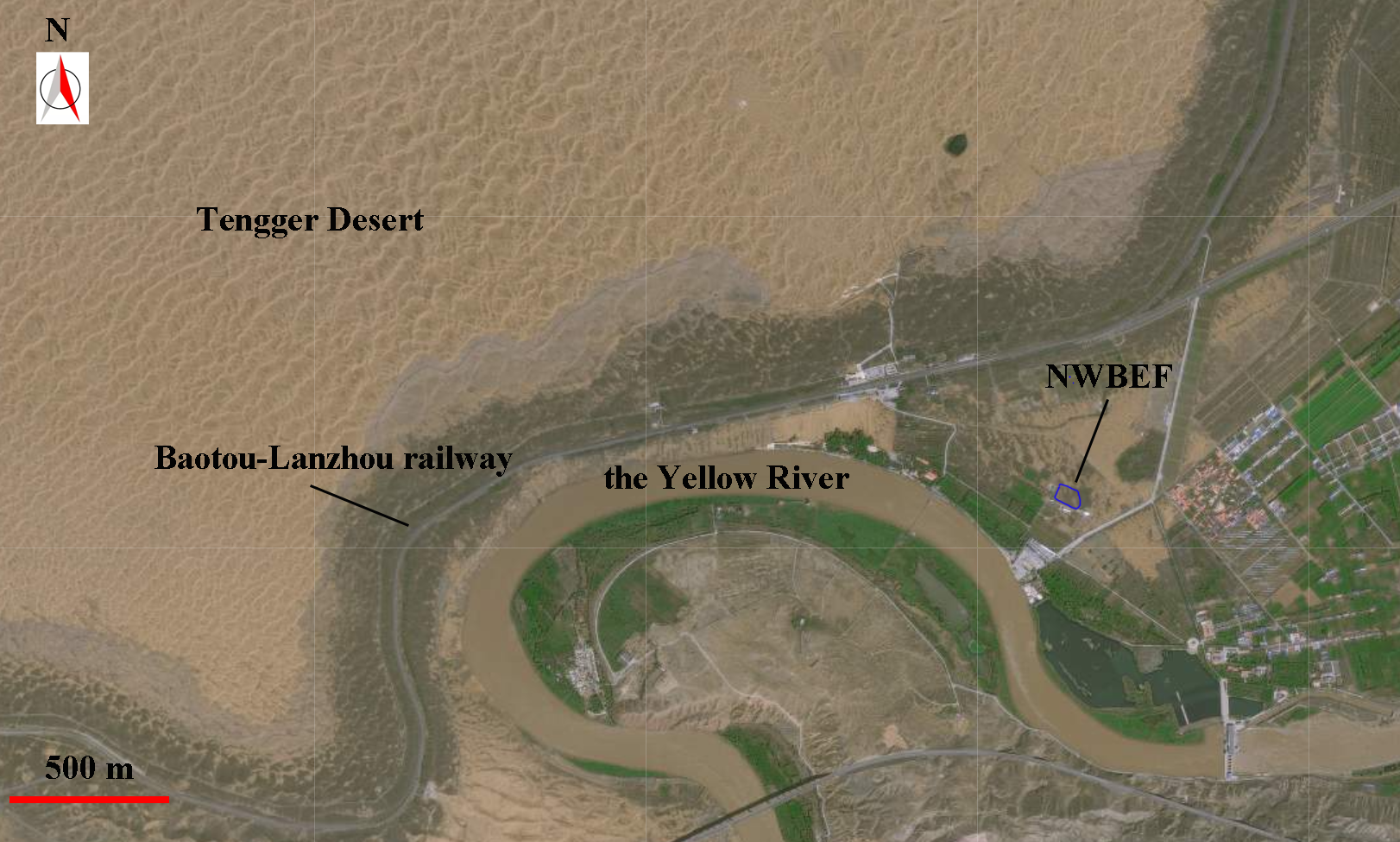


**Supplementary Figure 1.** Sketch map of the study area and aerial view of the NWBEF.


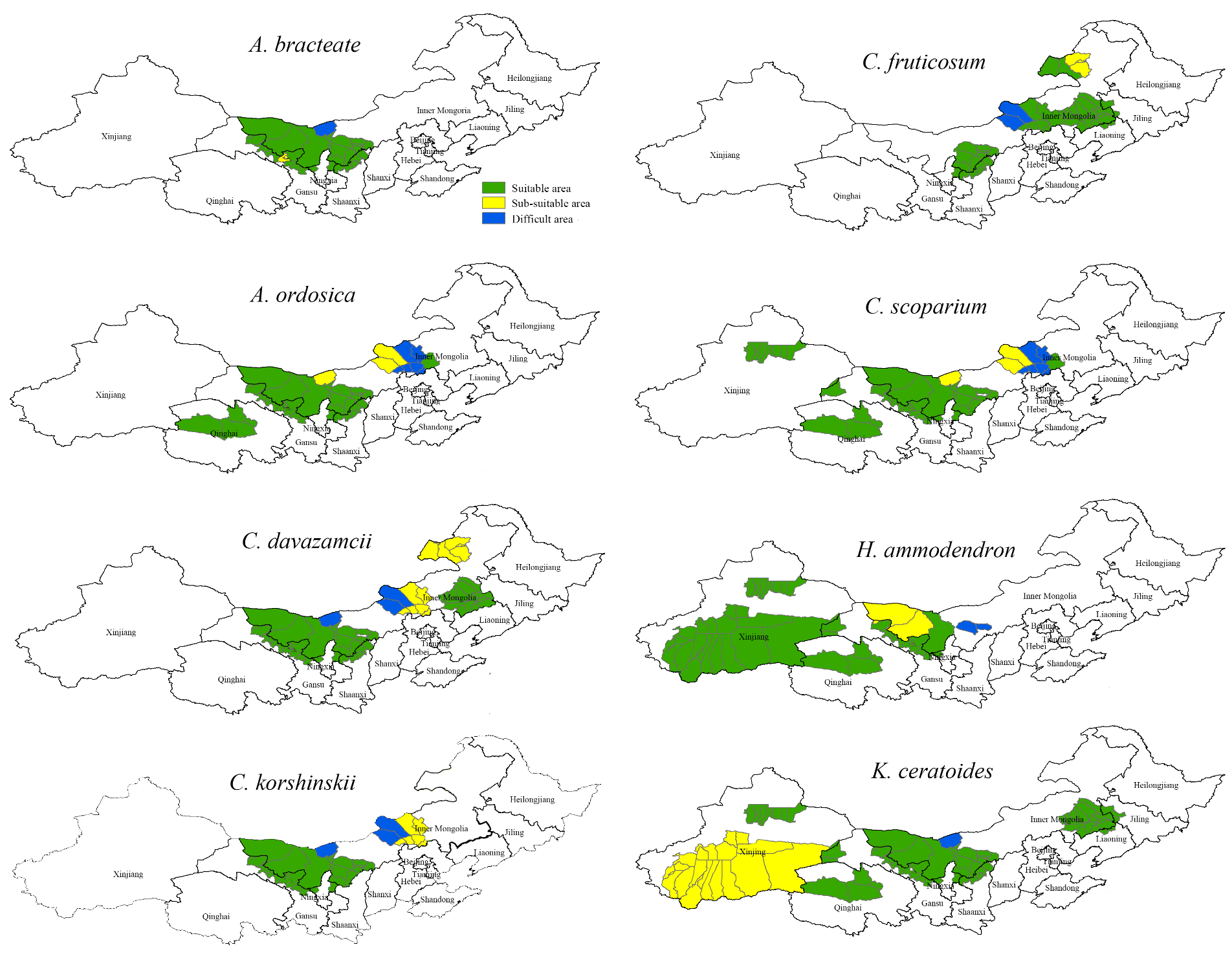


**Supplementary Figure 2.** Distribution of eight desert shrub species in the Northern China.





**Supplementary Figure 3.** Soil water content (a), precipitation (b) and percentage of annual precipitation (c) in the experimental plot during the test period.





**Supplementary Figure 4.** Vulnerability curves (VCs) of leaf for eight desert shrub species. VCs were plotted as *K_ls_* against Ψ_o_ using 40-45 shoots per species. The water potential corresponding to 50% of maximum leaf hydraulic conductance (*K_max_*, MPa) was determined as leaf P_50_ which was calculated by fitting a three-parameter sigmoidal regression function of the form: y = a ⁄ [1 + e^-k(x-xc)^], to the *K_leaf_* vs Ψ_o_ data (Blackman et al., 2010; Johnson et al., 2018).



 **Supplementary Figure 5.** Differences in traits among different genera. (a, b) the predawn and midday water potential (Ψ_pd_, Ψ_md_); (c) Water potential at 50% loss of maximum leaf hydraulic conductance (P_50_); (d) the difference between Ψ_pd_ and Ψ_md_; (e) maximum leaf hydraulic conductance (*K_max_*); (f and g) leaf hydraulic safety margins at 50% loss of conductivity (LSM_eo_) and hydraulic safety margins at turgor loss point (LSM_lw_); (h and i) Leaf hydraulic capacitance of pre-turgor loss point and post-turgor loss point (*C_pre-tlp_* and *C_pos-tlp_*); (j) Total leaf hydraulic capacitance (*C_total_*); (k) Osmotic potential at saturation (Ψ_π, sat_); (l) Water potential at turgor loss (Ψ_tlp_). All values are showed as mean ± standard error (SE), and different letters indicate statistically significant differences (One-way ANOVA, n = 5, *p* < 0.05).





**Supplementary Figure 6.** Pressure–volume (P-V) curves of leaf for eight desert shrub species. P-V curves were established following Nardini *et al.* (2013).





**Supplementary Figure 7.** Scree plot of the Principal Component Analysis (PCA) of the functional traits measured in 8 desert shrub species. All variables were equally weighted in the analysis.


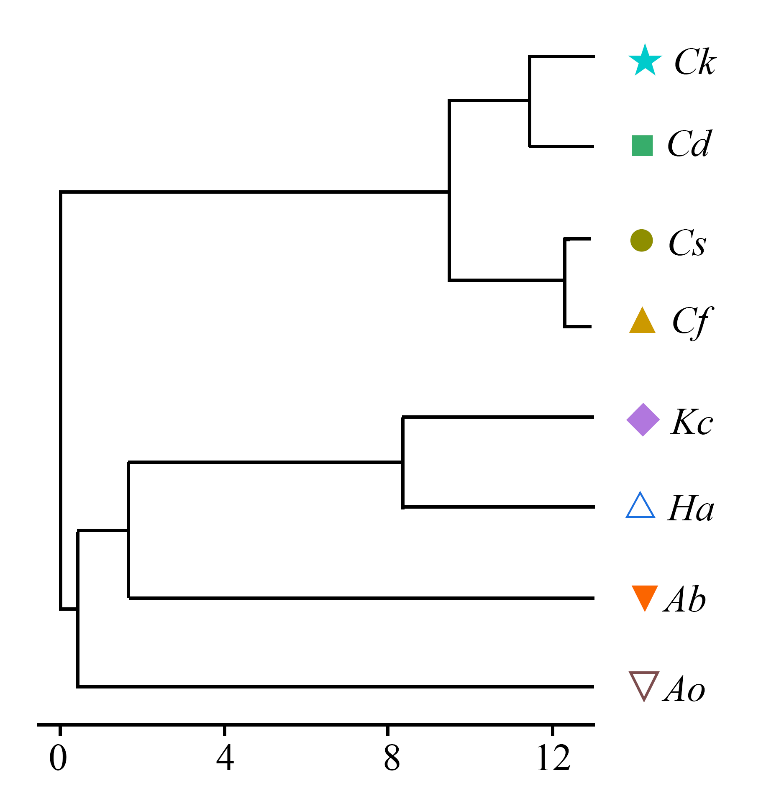


**Supplementary Figure 8.** Phylogenetic tree of 8 desert shrubs. *Atraphaxis bracteate* (*Atb*), *Artemisia ordosica* (*Aro*), *Caragana davazamcii* (*Cad*), *Caragana korshinskii* (*Cak*), *Corethrodendron fruticosum* (*Cof*), *Corethrodendron scoparium* (*Cos*), *Haloxylon ammodendron* (*Haa*), *Krascheninnikovia ceratoides* (*Krc*).





**Supplementary Figure 9.** The relationship between shrubs and leaf morphological traits and leaf hydraulic traits. All values are shown as mean ± SE. Shades of red represent 90% confidence intervals. Plant height (*H_p_*), crown width (*C_w_*), leaf area (*LA*), leaf length (*L_l_*), leaf width (*W_l_*), maximum leaf hydraulic conductance (*K_max_*), water potential at 50% loss of maximum leaf hydraulic conductance (P_50_), leaf hydraulic safety margins at 50% loss of conductivity (LSM_eo_), daily maximum water potential difference (*Δ*Ψ), osmotic potential at saturation (Ψ_π, sat_), relative water content at turgor loss (RWC_tlp_), bulk tissue modulus of elasticity (*ε*), leaf hydraulic capacitance of pre-turgor loss point (*C_pre-tlp_*).





**Supplementary Figure 10.** The relationship between shrubs and leaf morphological traits and leaf drought tolerance traits. All values are shown as mean ± SE. Plant height (*H_p_*), crown width (*C_w_*), leaf area (*LA*), leaf length (*L_l_*), leaf width (*W_l_*), maximum leaf hydraulic conductance (*K_max_*), water potential at 50% loss of maximum leaf hydraulic conductance (P_50_), leaf hydraulic safety margins at 50% loss of conductivity (LSM_eo_), daily maximum water potential difference (*Δ*Ψ), osmotic potential at saturation (Ψ_π, sat_), relative water content at turgor loss (RWC_tlp_), bulk tissue modulus of elasticity (*ε*), leaf hydraulic capacitance of pre-turgor loss point (*C_pre-tlp_*).

**1.2 Supplementary Tables**

**Supplementary Table 1.** The morphological traits and distribution of eight desert shrub species.

| Species | Family | *Abbreviation* | Morphological traits | Distribution |
| --- | --- | --- | --- | --- |
| *Atraphaxis bracteata* Losinsk. | Polygonaceae | *Atb* | Shrubs; height 1-1.5 m, the main stem is stout, light brown, glabrous, ribbed, and multi-branched; the leaf is leathery, oblong, or oval, 1.5-3.5 cm long and 0.8-2 cm wide. | Inner Mongolia, Ningxia, Gansu, Shaanxi. |
| *Artemisia ordosica* Krasch. | Compositae | *Aro* | Subshrub; height 0.5-1 m, the main root is thick, with many lateral roots; the rhizome is thick; the old branches are dark gray or grayish brown; the leaves are yellow-green, semi-succulent, the lower part of the stem leaves broadly ovate or ovate, one to two pinnately split. | Inner Mongolia, Gansu, Hebei, Shanxi, Shaanxi, Ningxia, Xinjiang. |
| *Caragana davazamcii* var. *davazamcii* | Fabaceae | *Cad* | Shrub; height 0.3 -1.5 (2) m, young branches are pilose. Pinnately compound leaves have 3-8 pairs of leaflets, the leaflets are elliptical and form an obovate ellipse, 3-10 mm long and 4-6 mm wide, densely pilose on both sides. | Inner Mongolia, Shaanxi, Ningxia, Gansu. |
| *Corethrodendron fruticosum* (Pall.) B. H. Choi & H. Ohashi | Fabaceae | *Cof* | Shrub; height 1-1.5 m, the young stems are green, the old stems are gray-white, the bark is longitudinally split, and the stems are more branched. | Liaoning, Inner Mongolia, Ningxia, Shaanxi. |
| *Caragana korshinskii* Kom. | Fabaceae | *Cak* | Shrub; height 1-4 m, tender branches are white pilose; pinnately compound leaves with 6-8 pairs of leaflets; leaflets are lanceolate or narrowly oblong, 7-8 mm long and 2-7 mm wide, gray-green, densely covered with white pilose on both sides. | Gansu, Inner Mongolia, Ningxia. |
| *Corethrodendron scoparium* (Fisch. & C. A. Mey.) Fisch. & Basiner | Fabaceae | *Cos* | Shrub; height 0.8-3 m, stems erect, young branches green or light yellow-green, stem bark is bright yellow, exfoliated in fibrous form; the lower part of the stem has leaflets 7-11, the upper leaf usually has leaflets 3-5, small leaves are gray-green, linear oblong or narrowly lanceolate. | Xinjiang, Qinghai, Gansu, Inner Mongolia, Ningxia. |
| *Haloxylon ammodendron* (C. A. Mey.) Bunge | Amaranthaceae | *Haa* | Big shrubs; height 1-9 m, the bark is gray-white; the old branches are gray-brown or light yellow-brown, leaf scales, broad triangle, slightly spread, blunt apex, axillary with cotton hair. | Ningxia, Gansu, Qinghai, Xinjiang, Inner Mongolia. |
| *Krascheninnikovia ceratoides* (L.) Gueldenst. | Amaranthaceae | *Krc* | Subshrub; height 0.1-1 m, branches are mostly concentrated in the lower part; branches are mostly concentrated in the lower part; the leaves are small, strip, strip-lanceolate, lanceolate, or oblong, 1-2-(5) cm long and 0.2-0.5-(1) cm wide. | Gansu, Inner Mongolia, Qinghai, Xinjiang, Tibet. |

Notes: Morphological traits and distribution of species comes from Chinese Virtual Herbarium (<https://www.cvh.ac.cn/index.php>).

**Supplementary Table 2.** The leaf traits of eight desert shrub species

|  | *L_l_* (cm) | *L_w_* (cm) |
| --- | --- | --- |
| *A. bracteata* | 3.12 ± 0.061e | 2.35 ± 0.079a |
| *A. ordosica* | 4.11 ± 0.084d | 2.23 ± 0.128a |
| *C. davazamcii* | 4.09 ± 0.138d | 1.31 ± 0.047b |
| *C. korshinskii* | 3.54 ± 0.103de | 1.14 ± 0.056b |
| *C. fruticosum* | 12.9 ± 0.496b | 2.46 ± 0.172a |
| *C. scoparium* | 7.80 ± 0.409c | 2.31 ± 0.157a |
| *H. ammodendron* | 17.5 ± 0.578a | 0.116 ± 0.003c |
| *K. ceratoides* | 2.87 ± 0.139e | 0.255 ± 0.016c |

**Supplementary Table 3.** Loadings of the functional traits of the first two principal components in the Principal Component Analysis (PCA).

| Trait | PC1 | PC2 | PC3 |
| --- | --- | --- | --- |
| Ψ_pd_ | **0.292** | 0.046 | 0.092 |
| Ψ_md_ | **0.344** | 0.094 | 0.106 |
| *△*Ψ | **-0.336** | -0.099 | -0.103 |
| *K_max_* | 0.168 | 0.244 | **0.283** |
| P_50_ | 0.265 | -0.091 | **0.375** |
| *H_p_* | 0.155 | -0.173 | **0.191** |
| *C_w_* | 0.101 | -0.006 | **0.324** |
| *LA* | **0.225** | -0.110 | 0.0682 |
| Ψ_π, sat_ | **-0.343** | 0.134 | 0.180 |
| Ψ_tlp_ | -0.073 | 0.021 | **0.491** |
| RWC_tlp_ | 0.147 | **-0.351** | -0.201 |
| -1/*b* | 0.244 | **0.284** | 0.059 |
| -1/*β* | **0.214** | 0.044 | -0.020 |
| *ε* | 0.159 | **-0.339** | -0.289 |
| LSM_eo_ | 0.217 | **0.299** | -0.256 |
| LSM_lw_ | **0.319** | 0.101 | -0.274 |
| *C_pre-tlp_* | -0.249 | **0.332** | 0.009 |
| *C_pos-tlp_* | 0.106 | **0.383** | -0.208 |
| *C_total_* | -0.058 | **0.417** | -0.130 |
| Eigenvalue | 5.81 | 4.81 | 2.55 |
| Percentage of Variance (%) | 30.6 | 25.3 | 13.4 |
| Cumulative (%) | 30.6 | 55.9 | 69.3 |

Notes: *K_max_*, the maximum leaf hydraulic conductance; Ψ_pd_, the predawn water potential; Ψ_md_, midday water potential; *△*Ψ, the difference between Ψ_pd_ and Ψ_md_; P_50_, the water potential corresponding to 50% of *K_max_*; *H_p_*, plant height; *C_w_*, Crown width; *LA*, Leaf area; Ψ_π, sat_, the osmotic potential at saturation; Ψ_tlp_, water potential at turgor loss point; RWC_tlp_, relative water content at turgor loss point; -1/*b* and -1/*β*, water sensitivity coefficients before and after turgor loss point; *ε*, bulk tissue modulus of elasticity; LSM_eo_ and LSM_lw_, Leaf hydraulic safety margins at 50% loss of conductivity and at turgor loss point; *C_pre-tlp_* and *C_pos-tlp_*, capacitance of pre- and post-turgor loss point; *C_total_*, total capacitance.
